# Development of a composite outcome score for a complex intervention - measuring the impact of Community Health Workers

**DOI:** 10.1186/s13063-015-0625-1

**Published:** 2015-03-21

**Authors:** Hilary Watt, Matthew Harris, Jane Noyes, Rhiannon Whitaker, Zoe Hoare, Rhiannon Tudor Edwards, Andy Haines

**Affiliations:** Department of Primary Care and Public Health, Imperial College London, Reynolds Building, St Dunstans Road, London, W6 8RP, England UK; School of Social Sciences, Bangor University, Bangor, Wales UK; London School of Hygiene and Tropical Medicine, London, England UK; NWORTH Clinical Trials Unit, Bangor University, Bangor, Wales UK; Centre for Health Economics and Medicine Evaluation, Bangor University, Bangor, Wales UK; Whitaker Research Ltd., London, UK

**Keywords:** Health services research, Health impact assessment, Delivery of health care, Research outcomes

## Abstract

**Background:**

In health services research, composite scores to measure changes in health-seeking behaviour and uptake of services do not exist. We describe the rationale and analytical considerations for a composite primary outcome for primary care research. We simulate its use in a large hypothetical population and use it to calculate sample sizes. We apply it within the context of a proposed cluster randomised controlled trial (RCT) of a Community Health Worker (CHW) intervention.

**Methods:**

We define the outcome as the proportion of the services (immunizations, screening tests, stop-smoking clinics) received by household members, of those that they were eligible to receive. First, we simulated a population household structure (by age and sex), based on household composition data from the 2011 England and Wales census. The ratio of eligible to received services was calculated for each simulated household based on published eligibility criteria and service uptake rates, and was used to calculate sample size scenarios for a cluster RCT of a CHW intervention. We assume varying intervention percentage effects and varying levels of clustering.

**Results:**

Assuming no disease risk factor clustering at the household level, 11.7% of households in the hypothetical population of 20,000 households were eligible for no services, 26.4% for 1, 20.7% for 2, 15.3% for 3 and 25.8% for 4 or more. To demonstrate a small CHW intervention percentage effect (10% improvement in uptake of services out of those who would not otherwise have taken them up, and additionally assuming intra-class correlation of 0.01 between households served by different CHWs), around 4,000 households would be needed in each of the intervention and control arms. This equates to 40 CHWs (each servicing 100 households) needed in the intervention arm. If the CHWs were more effective (20%), then only 170 households would be needed in each of the intervention and control arms.

**Conclusions:**

This is a useful first step towards a process-centred composite score of practical value in complex community-based interventions. Firstly, it is likely to result in increased statistical power compared with multiple outcomes. Second, it avoids over-emphasis of any single outcome from a complex intervention.

## Background

There are many challenges in designing studies of complex interventions [1], one of which is to decide upon appropriate outcome measures. When undertaking a randomised trial, and other evaluation studies, it is desirable to find a single primary outcome measure so that statistically robust conclusions about the success of the intervention can be made. Meta-analysis then becomes possible in a review of similar complex intervention effects [2]. When the intervention has a wide variety of potential outcomes it is much harder to identify the single primary outcome of choice. Selecting one outcome over several possible alternatives may distort the overall purpose of the trial [3]. An alternative may be to collect many different measures to assess the effectiveness of the intervention, yet this may require statistical adjustments such as the Bonferroni correction [4].

In clinical trials, composite outcome scores have become widely used where multiple possible outcomes may arise from an intervention [5-7]. ‘All-cause mortality’ or ‘time to treatment failure’ are composed of multiple individual outcomes and, when well-combined, defined and reported [8], have the benefits of increasing statistical precision of the trial, without resorting to an arbitrary selection of one primary outcome over another [9].

However, in health services research or where complex interventions are proposed, it may be more useful to measure changes in the delivery of care. Measurement of changes to hard clinical outcomes may be beyond the scope of a trial. In such instances, there may be many potential process outcomes of interest. There is little if any guidance, however, on how to develop a composite process indicator for a complex intervention. Guidance on process evaluations of complex interventions, such as the recent comprehensive guidance from the Medical Research Council [10], focus on mixed methods research to capture the quantitative and qualitative aspects of a change process. It would, however, be useful to be able to develop a composite score of process outcomes, such as service utilisation, particularly in trial situations.

The use of Community Health Workers (CHWs) is one such example of an intervention that can have many potential process outcomes of interest. CHWs are defined as members of the local community, who are engaged in local health promotion activities, yet who have less training than other health professionals such as nurses. The World Health Organisation (WHO) promotes their use globally and particularly in developing countries, in response to a lack of fully trained medical professionals [11].

We discuss here how the impact of CHWs (and other interventions with a similar intention) might be evaluated using a novel composite process outcome indicator. In our example, we assume that CHWs would visit all households (in their allocated geographical area and registered with the GP to whom the CHW reports) on a monthly basis. Their remit would be to offer a comprehensive and wide range of health support and advice, to each member of the household at least once per month irrespective of expressed need or demand - consistent with a similar model that is working successfully on a large scale in Brazil [12-20]. For such an intervention, we would expect there to be a wide number of potential outcomes ranging from improving uptake of screening and immunisation services to promoting healthy behaviours. One goal would be to measure the potential of the proposed CHW to increase the uptake of immunisation, screening services and other services that are currently available on the National Health Service (NHS). In the context of a trial, it would be necessary to identify a suitable primary outcome measure. However, as the CHWs deliver advice and support across a wide range of health domains and age groups it is impossible to identify the single primary outcome measure of choice without distorting the purpose of an eventual trial. Furthermore, an intervention such as this, focusing particularly on increasing the uptake of preventive interventions, is unlikely to have an effect on hard clinical outcomes within the duration of a feasibility study. Process measures such as uptake of services are an interesting alternative because they help explain how clinical changes come about and, in the case of this proposed community-based service, are closer to the level of the intervention. However, there may be several process outcomes of equal interest and so one option may be to construct a composite outcome score to capture as much of the total effect of the CHW as possible.

We have been exploring the possibility of embedding such a service into local GP practices in deprived communities in North Wales [21]. It is in this context that we describe the rationale and analytical considerations for this process (rather than clinical) composite outcome score. We simulate its use in a large hypothetical population, and use it to calculate sample sizes for a suitably powered cluster randomised controlled trial (RCT) in this context. Our proposed composite primary outcome, which we call the Composite Referral Completion Indicator (CRCI), for evaluation of the CHW intervention reflects uptake rates of a number of screening tests and immunisations and attendance at stop-smoking clinics. This outcome is defined at the household level, which corresponds to the level at which the CHW intervention is delivered. It evaluates the success with which the CHW is able to improve the uptake of services by household members that are eligible for those services. This article may serve as a useful example of how a process composite score may be designed around a complex intervention, and may help others to find appropriate outcomes for their own purposes.

## Methods

We define the CRCI as the proportion of the services received by household members, of those that they were eligible to receive. It is calculated as the number of scheduled (or later than scheduled) screening tests and/or scheduled immunisations received by household members plus number of people who attend stop-smoking clinics divided by the number of screening tests and/or immunisations and/or stop-smoking clinics that household members are eligible for during the study period. For instance, a household might consist of one single woman who, based on her age, is eligible only for one service - the cervical smear test. If she receives this test, within the designated time period of the study, then the CRCI would be calculated to be one (one divided by one, that is full uptake of the service). If she did not take up the screening then it would be calculated to be zero (zero divided by one). In an alternative household where there is a child who is eligible only for the influenza immunisation, a mother who is eligible for both breast cancer and cervical screening, and a father who is not eligible for anything, the denominator for the CRCI would be three. If only one of these services is taken up during the study period then the CRCI would be calculated to be 1/3.

We define a household as one or more people living in one postal address. We define a household member as anyone ordinarily resident in the household, including babies born to the household during the study. We define a household member to be eligible for a service if they meet the standard criteria for them to be eligible for a service provided on the NHS. These criteria are usually based on age and sex, established for each individual screening or immunisation programme during the study period. Equally, we define uptake of a service if this has occurred anytime during the study period and up to 3 months after the study period. It is possible to extend the CRCI definition to include household members who move in and out, by including household members’ service needs into the denominator, scaled according to the proportion of the study period that they live in the household, and by including their met service needs into the numerator. The simulation exercise does not include such complexities, since allowance for them is unlikely to appreciably affect the power. Table 1 shows the immunisations, screening procedures and other services currently offered on the NHS and the uptake rates currently achieved in North Wales, where our feasibility work took place. We list the services according to their age and sex criteria. It further shows the proportion of households who will have a member in each age/sex group. This sets out the potential for improvement, which will be measured by the CRCI, and so is a starting point for assessing realistic estimates to put into sample size calculations.

**Table 1:** **Eligibility for screening** ^**a**^ **and immunisation** ^**a**^ **procedures and smoking cessation clinic on the National Health Service (NHS), with uptake rates currently achieved in North Wales**

**Household member type**	**Population** ^**b**^ **distribution by age/sex group**	**Proportion** ^**c**^ **of HHs with a member of this type**	**Service 1**	**Uptake % (service 1)**	**Service 2**	**Uptake % (service 2)**	**Service 3**	**Uptake % (service 3)**	**New service**
Baby 0 to 11 months	1.2%	2.8%	DPT/Hib	96.9	Pneumococcal	96.6	Meningococcal	96.6	Rotavirus
Infant 12 to23 months	1.3%	3.0%	Pneumococcal	94.3	Hib/MenC	94.5	MMR	93.5	
Infant 2 years	1.3%	3.0%							Influenza
Infant 3 years	1.3%	3.0%	DPT/polio	92.3	MMR	93.5			Influenza
Child 4 to 15 years	13.5%	23.3%							Influenza
(includes girls 13/14 years)	(1.1%)	(2.8%)	HPV	83.4					
Youth 16 to 19 years	4.7%	8.6%							Chlamydia^**b**^
Female 20 to 25 years	3.8%	7.9%	Cervical^**b**^	76.5					Chlamydia^**b**^
Female 26 to 49 years	17.2%	39.9%	Cervical^**b**^	76.5					
Female 50 to 59 years	6.1%	13.6%	Cervical^**b**^	76.5	Breast^**b**^	73.3			
Female 60 to 64 years	2.9%	6.7%	Cervical^**b**^	76.5	Breast^**b**^	73.3	Bowel^**b**^	54.0	
Female 65 to 70 years	2.8%	6.6%	Breast^**b**^	73.3	Bowel^**b**^	54.0	Influenza	68.2	Varicella
Female 71 to 74 years	1.7%	3.9%	Influenza	68.2					
Female 75 years plus	4.6%	10.5%	Influenza	68.2					
Male 20 to 25 years	3.6%	7.1%							Chlamydia^**b**^
Male 26 to 59 years	22.6%	49.9%							
Male 60 to 64 years	2.8%	6.2%	Bowel^**b**^	54.0					
Male 65 to 70 years	2.9%	6.7%	Bowel^**b**^	54.0	Influenza	68.2			
Male 71 to 74 years	1.4%	3.2%	Influenza	68.2					Varicella
Male 75 years plus	3.1%	7.2%	Influenza	68.2					
Pregnant women	1.3%	3.0%	Influenza	43.1					
Diabetics over 12 years	2.8%	6.5%	Retinal^**b**^	81.0					
At high risk (for example, CVD)	4.5%	10.3%	Influenza	52.0					
Smokers of any age	19.0%	38.2%	Smoking cessation	9.0					

^**a**^Services included in the Composite Referral Completion Indicator (CRCI), according to age and sex, based on England and Wales uptake rates currently achieved.

^b^Population distribution within each age and sex band, based on England and Wales population structure^a^.

^c^Proportion of households that have one or more members within each specified age/sex band, based on England and Wales population structure^a^.

*Abbreviations*; *CVD* cardiovascular disease, *HH* households.

The CRCI outcome measure is calculated for each household and can be analysed as a proportion. In order to find the characteristics (mean and standard deviation) of the CRCI for use in sample size calculations, we simulated the characteristics of the CRCI against a hypothesised population. In order to do this, we firstly find the age/sex distribution within households, in order to apply the uptake of service information provided in Table 1. The age/sex distribution of households is not available from routine data without complex triangulation between multiple data sources in primary and social care, so we have simulated a population household structure (by age and sex), based initially on household composition data from census data from England and Wales.

We simulated the distribution of households by number of occupants and by the age and sex mix within each household. This was based on the distribution of household types [22] and number of occupants by household type [23] in England and Wales as reported by the Office for National Statistics (ONS), which divides household types into those which are single occupancy, married couples, same-sex civil partnerships, cohabiting couples and lone parents, student households and other households. Cohabiting couples were distributed amongst married couples and same-sex civil partnerships [24]. Some of these categories are further divided by ONS into those with dependent children (one child, two or more children), only non-dependent children and no children. Some categories (for example, single occupancy) are also divided by ONS into those over and below 65 years (all above this age for multiple occupancy household). Ages of parents of children were derived from ages of parents on giving birth [25]. Relative ages of siblings were made to fit in with ONS data on distribution of ages of youngest child, by number of dependent children [26]. Sex distributions of lone parents and of same-sex civil partnerships were derived from published ONS data [27]. Age and sex distribution of the simulated population was made to match with the age and sex distribution of England and Wales [28]. The above process resulted in a distribution of households of varying sizes from single occupancy to containing eight occupants (the maximum size that we considered), by age band and sex. A random sample of 20,000 households was then selected, sampled according to frequency of different household constitutions by age and sex.

We then applied the characteristics of smoking, diabetes and pregnancy by age and sex group, since there are defined interventions for each of these groups. A simple random process was used to determine who in our random sample of individuals within households has each characteristic. Eligibility to different screening tests, immunisations or services was allocated based on the current criteria for each separate service [29-37].

The rates of diabetes, smoking, and of being in an ‘at risk’ category for the seasonal influenza immunisation, were based on reported rates by age and sex [38]. Smoking rates are taken from Cancer Research UK statistics [39]. Pregnancy rates were assumed to be constant amongst married couples, as well as allowing for some pregnancies amongst dependent teenagers; overall rates are set to equal birth rates in England and Wales [25] and allowing for 12 months of pregnancies to be included at any specified gestational age. These characteristics were allocated to individuals within households by random Bernoulli distributions, according to eligibility and prevalence in each age and sex group - with diabetics always classified as ‘at risk’ for the seasonal influenza immunisation. The uptake rates of screening tests and immunisations were based on reported uptake rates for Wales, or North Wales, as available [40].

For the purposes of this simulation, we took the study to have 1 year of follow-up and based the eligibility for interventions within each age/sex group on this 1-year period. For immunisations, those within the appropriate age ranges are taken to be eligible for the intervention. For adult screening programmes, some services are available only every 2 or 3 years and allowance is made for the provision to make up for missed screening tests. Assuming uptake is evenly distributed during the period that the service is available, the proportion of the age/sex group eligible during any single year is a third (for 3-yearly screening, or a half for 2-yearly), with an allowance for the proportion who are just coming into the age range (who are all eligible), plus a proportion of the remainder who did not previously attend (1 − uptake rate).

Uptake rates of the different screening tests and immunisations were then used to simulate how many of the eligible people had taken up these opportunities within households, based on random sampling from the binomial distribution with the reported uptake rates as the population proportion. This gives the anticipated uptake rates in the control arm of the CHW intervention study. For the treatment arm (with the CHW intervention), we simulated that 10%, 20% and 30% of those that had not taken up the service take them up because of the CHW home visit (that is the initial reported uptake rate, proportion r, is increased to uptake rate r + 0.1 × (1 − r) for a 10% intervention percentage effect). Again random sampling from the binomial distribution with these new uptake rates is applied to find the distribution of individuals within households who have and have not taken up the interventions (out of any eligible).

The CRCI ratio was calculated for each household. The denominator is the total number of services that the specified household is eligible for, summed across all service types (listed in Table 1), with duplicate counting of any that more than one person is eligible for. The numerator is the total number of these services taken up during the study period, and so must be between zero (for households who do not comply with anything) and the denominator (for fully compliant households). There are some households who are not eligible for any services. The CRCI is undefined for them (with numerator and denominator of zero).

The mean and standard deviation of the CRCIs (measured at household level) are computed in our random sample of 20,000 households, for the control arm (based on current uptake rates) and for the CHW intervention arms (with various levels of effectiveness). These are used to calculate sample sizes required for an individually RCT of the CHW intervention, using standard formulae for comparison of two means [4] adjusted for the design effect and intra-class correlation coefficient (ICC) [4].

Various sensitivity analyses were then undertaken as follows. We examined the effect of removing some of the screening/immunisations and we estimated the effect of the disease risk factors (smoking, diabetes and ‘at risk’ re influenza immunisation) being very similar within households, rather than being randomly distributed amongst households. We also explored the effect of assuming that 50% of households were generally adherent and 50% generally non-adherent to the intervention. The uptake rate in the compliant households was taken to be over 99% (more precisely uptake rate, r, was increased to r + 0.99 × (1 − r)), and in less compliant households, the uptake rate was reduced by the same amount (to r − 0.99 × (1 − r), with a modification for rates < 50%). The effect of undertaking this intervention in a deprived area was also assessed by using the distribution of households with the highest proportion of lone parent households (Barking and Dagenham in Outer London). This study did not require ethical approval.

## Results

Table 1 shows the different screening tests and immunisations that people are eligible for on the NHS, for inclusion in CRCI outcome, according to age and sex. Most age/sex groups are eligible for at least one intervention, and hence the CRCI will be defined on any households containing them. The main exception is middle-aged men, who are only eligible for an intervention if they smoke, have diabetes or are otherwise considered high-risk and in need of the seasonal flu vaccination. Hence the CRCI is not always defined on a household that contains only one or a few middle-aged men. Some age/sex groups are eligible for more than one service, such as infants and middle-aged women scheduled for cancer screening tests. The table also shows the proportions of the simulated population (based on the England and Wales population structure) that fall within each age/sex group, and the proportions of households that are comprised by each specified age/sex group. This defines the potential for improvement in rates, as evaluated by the CRCI outcome measure.

Table 2 shows the distribution of households by household type according to census definitions and the distribution of the number of services for inclusion in the CRCI outcome that the household members are eligible for per household type. This is effectively a step *en route* to calculation of the sample size requirements. It is based on our determination of the detailed breakdown of household sizes and age/sex distributions within them, found by modelling census data, including on relative ages of married couples, and on ages of mothers and fathers at birth of their children, plus other census information as detailed in the methods section. We considered all services, immunisations and screening tests from Table 1, except chlamydia (since this is confidential information and unlikely to be obtainable in practice). Vaccine programmes such as rotavirus and varicella that have not yet been introduced but those that will be in the near future have been given estimated uptake rates based on similar vaccine programmes. Using the simulated household characteristics modelled from the census, and the reported uptake rates for each service (except chlamydia) and assuming no disease risk factor clustering at the household level, then overall 11.7% of households are eligible for no services, 26.4% for 1, 20.7% for 2, 15.3% for 3 and 25.8% for 4 or more.

**Table 2:** **Service eligibility for services included in the Composite Referral Completion Indicator (CRCI) by household type based on England and Wales population structure** ^**a**^

**Household type**	**Prevalence**	**Proportion according to degree of service eligibility**				
		**No services**	**One service**	**Two services**	**Three services**	**≥ Four services**
Single person (16 to 64)	17.59%	43.3%	36.1%	14.2%	5.1%	1.3%
Single person (65 plus)	12.49%	0.0%	75.2%	21.4%	3.2%	0.3%
Married^b^ (at least one < 65), no children	17.36%	13.4%	30.1%	26.4%	16.2%	13.9%
Married^b^ (65 plus), no children	8.17%	0.0%	0.0%	24.7%	27.9%	47.3%
Married^bc^ with one dependent child	8.34%	1.1%	13.3%	27.6%	26.3%	31.7%
Married^bc^ with two or more dependent children	12.04%	0.4%	3.2%	9.1%	17.7%	69.7%
Married^c^ with only non-dependent child(ren)	6.25%	3.9%	13.6%	20.9%	22.3%	39.3%
Lone mothers with one dependent child	3.44%	1.9%	20.2%	35.6%	21.8%	20.5%
Lone mothers with two or more dependent children	2.72%	0.0%	4.4%	14.5%	19.3%	61.7%
Lone mothers with only non-dependent child(ren)	2.10%	9.5%	24.5%	29.8%	19.0%	17.1%
Lone fathers with one dependent child	0.43%	7.0%	38.4%	27.9%	12.8%	14.0%
Lone fathers with two or more dependent children	0.35%	1.4%	5.8%	17.4%	27.5%	47.8%
Lone fathers with only non-dependent child(ren)	1.34%	23.2%	33.7%	27.0%	12.0%	4.1%
Same-sex partnerships, no children	0.26%	27.5%	19.6%	33.3%	7.8%	11.8%
Same-sex partnerships with child(ren)	0.01%	0.0%	0.0%	0.0%	50.0%	50.0%
Other households (including extended families) with one dependent child	0.97%	0.0%	0.5%	8.2%	20.1%	71.1%
Other households (including extended families) with > one dependent child	0.87%	0.0%	0.0%	0.6%	6.4%	93.1%
Student households, all students	0.55%	10.1%	22.9%	23.9%	20.2%	22.9%
Other households (all 65 plus)	0.29%	0.0%	0.0%	21.1%	36.8%	42.1%
Other households (not all 65 plus)	4.48%	15.4%	21.9%	24.4%	16.3%	22.0%
TOTAL/Weighted average proportions	100.00%	11.7%%	26.4%%	20.7%	15.3%	25.8%

^a^Excluding chlamydia services, assuming disease risk factors are not clustered at household level.

^b^Married includes cohabiting couples of opposite sex. ^c^Some ‘other households with dependent children’ are incorporated within these categories.

The sample size needed to power a controlled intervention study is shown in Table 3. The estimates depend strongly on intervention percentage effect. We simulated the sample size requirements for when 10%, 20% and 30% of services, immunisations or screening procedures which otherwise would not have been taken up, are taken up as a result of the CHW intervention. To demonstrate a small CHW intervention percentage effect (10%) and assuming low intra-household clustering for disease risk factors and uptake of services, and low intra-CHW ICC, 1,650 households would be needed in each of the intervention and control arms - equating to 17 CHWs in the intervention arm. If the CHWs were more effective (30%), then only 170 households would be needed in each of the intervention and control arms to demonstrate this, which equates to only 1 or 2 CHWs in the intervention arm (though at least 5 to 10 would be needed for generalisability of findings).

**Table 3:** **Sample sizes for a controlled intervention study with Composite Referral Completion Indicator (CRCI) as the outcome measure, based on England and Wales population structure** ^**a**^

**Level of similarity within households in disease risk factors/level of similarly within households in level of compliance of uptake of interventions**	**Intervention percentage effect**	**Mean (SD) CRCI in control arm**	**Mean (SD) CRCI in CHW arm**	**Sample size number of households per arm based on comparison of mean CRCIs, allowing for inclusion of households** ^**b**^ **without valid CRCI:**			
				**Without clustering of intervention by region**	**With clustering of intervention by regions of 100 households, Intra-CHW ICC = 0.01**	**With clustering of intervention by regions of 100 households, Intra-CHW ICC = 0.02**	**With clustering of intervention by regions of 100 households, Intra-CHW ICC = 0.05**
Typical distribution across England and Wales: none/none	10%	0.56 (0.36)	0.61 (0.36)	1,650	3,280	4,920	9,820
none/none	20%	0.56 (0.36)	0.65 (0.35)	400	800	1,190	2,380
none/none	30%	0.56 (0.36)	0.69 (0.34)	170	340	510	1,010
none/high	10%	0.56 (0.41)	0.61 (0.39)	1,970	3,920	5,870	11,720
none/high	20%	0.56 (0.41)	0.65 (0.38)	490	980	1,460	2,920
none/high	30%	0.56 (0.41)	0.69 (0.36)	210	420	630	1,250
high/none	10%	0.6 (0.37)	0.64 (0.36)	1,990	3,960	5,930	11,840
high/none	20%	0.6 (0.37)	0.68 (0.35)	490	980	1,460	2,920
high/none	30%	0.6 (0.37)	0.72 (0.33)	210	420	630	1,250
high/high	10%	0.6 (0.41)	0.64 (0.39)	2,470	4,920	7,360	14,700
high/high	20%	0.6 (0.41)	0.68 (0.38)	590	1,170	1,760	3,510
high/high	30%	0.6 (0.41)	0.72 (0.36)	250	500	750	1,490
Typical distribution of deprived areas none/none	10%	0.57 (0.36)	0.61 (0.35)	1,650	3,280	4,920	9,820
none/none	20%	0.57 (0.36)	0.66 (0.34)	400	800	1,190	2,380
none/none	30%	0.57 (0.36)	0.7 (0.33)	170	340	510	1,010
none/high	10%	0.57 (0.4)	0.62 (0.39)	1,970	3,920	5,870	11,720
none/high	20%	0.57 (0.4)	0.66 (0.37)	480	960	1,430	2,860
none/high	30%	0.57 (0.4)	0.7 (0.36)	210	420	630	1,250
high/none	10%	0.6 (0.36)	0.64 (0.35)	2,010	4,000	5,990	11,960
high/none	20%	0.6 (0.36)	0.68 (0.34)	480	960	1,430	2,860
high/none	30%	0.6 (0.36)	0.72 (0.33)	210	420	630	1,250
high/high	10%	0.6 (0.4)	0.64 (0.39)	2,400	4,780	7,150	14,280
high/high	20%	0.6 (0.4)	0.68 (0.37)	580	1,150	1,730	3,450
high/high	30%	0.6 (0.4)	0.72 (0.35)	250	500	750	1,490

^a^(Excluding chlamydia) and based on 90% power at 5% significance level. ^b^88.3% of households have valid CRCI with no intra-household correlation of disease risk factors, and 84.8% of households have valid CRCI based on high intra-household correlation of disease risk factors. Intervention percentage effect is proportion of services (immunisations/screening tests, stop-smoking clinics) that are taken up with CHW intervention, out of those services that would not otherwise be taken up.

*Abbreviations*: *CHW* Community Health Worker, *ICC* intra-class correlation coefficient.

In deprived areas, there are far more single parent households (15% with one or more dependent children) than in England and Wales as a whole (8%). Other households containing dependent children also make up a slightly larger proportion of the total (that is the married/cohabiting group and particularly other households with dependent children). The number married/cohabiting without children is reduced (14% in the deprived community compared to 26% in England and Wales). Distribution of household types for a typically deprived area results in slightly higher proportions of households with measurable outcomes (89.2% rather than 88.3% without correlation of disease risk factors). This has a negligible effect on sample sizes (within 3% of those in Table 3).

We modelled the distribution of service eligibility with the extreme clustering of disease risk factors (smoking, diabetes and at-risk flu vaccine) within households (more extreme than would be found in practice). This results in fewer households that are eligible for any service (84.8%), and an increase in sample sizes requirements of 19 to 26%. Modelling strong clustering of uptake of services within households also results in an increase in sample sizes by around 19 to 24%. The intra-CHW ICC has a much greater effect on the sample size requirements. With an ICC of 0.01 and 100 households per CHW, sample size requirements are doubled, compared to if there was no intra-CHW clustering. They are trebled for an ICC of 0.02, and 6-fold for ICC of 0.05. For the greatest clustering throughout, with an intra-CHW ICC of 0.05 and high intra-household clustering (clustering of disease risk factors and uptake rates), then between 10,000 and 14,000 households (that is 100 to 140 CHWs) would be needed in both control and intervention arms to show a 10% intervention percentage effect, and 1,000 to 1,400 households in each arm (and 10 to 14 CHWs in total) would be needed to show a 30% intervention percentage effect.

## Discussion

We have modelled a composite process outcome score for a complex CHW intervention applied to a hypothesised population in North Wales. This modelling exercise could be applied in other scenarios. The steps we took were: (1) find distribution of eligibility to different aspects of the composite outcome score (at household level in our example since the outcome is defined at household level, but more typically at individual level), and simulate a large population for this purpose, (2) apply knowledge of the distribution of the different components of the outcomes in the population in the absence of any intervention, using random sampling from the Bernoulli distribution for each individual component of the outcome measures, (3) suggest plausible effectiveness of the intervention, and (4) apply knowledge of the distribution of the different components of the outcomes with the intervention, using random sampling from the Bernoulli distribution for each individual component of the outcome measure.

Using this approach we found that large sample sizes would be needed to undertake an intervention study basing the primary outcome on the CRCI, and yet these sizes are potentially achievable. For an intervention percentage effect of just 10%, around 2,000 households may be needed per arm. For an intervention percentage effect of 20% and of 30%, only a quarter and a tenth of the number of households is required.

It is noteworthy that the time when such an intervention study period is started should correspond to a time before the start of the flu season, and end after the end of the season, so that its effect on this vaccination can best be calculated. It would be appropriate to take an intervention period of at least a year (the time period used in this simulation). A period of 3 years may be preferable, since some cancer screening tests are scheduled every 3 years, but then it may not be desirable to wait too long before evaluating the result. The apparent effect of any such intervention may be different in the first year of its operation (when there may be more previously untapped demand to pick up), compared to when it has been running for some time.

Other CHW projects of a similar nature have elected to use single primary outcomes as a measure of the CHW impact, with other measures relegated to secondary importance [41]. However, the CHW project we have proposed is not an intervention for the improvement of, as examples, HbA1c or blood pressure measurements, alone. Rather it is an intervention to improve, broadly, the interface between the household and primary care - ensuring that individuals with expressed and unexpressed need or demand are appropriately referred to the correct service in a timely manner. For this, process measures such as service utilisation are needed, and the CRCI tells us whether the home-based CHW intervention improves overall service uptake and utilisation. This is reasonable because the intervention is likely to elicit an effect across all components of the composite score and there is causal relevance between the intervention and each component of the CRCI [42]. Uptake of a service may not only be the result of the CHWs’ efforts, and so the CRCI does need to be interpreted against a control group. The components of a composite outcome score should always be declared as secondary outcomes and described alongside the primary composite score [9].

In pharmaceutical trials, the components of a composite score are measurable events that can sensibly be added together as being aspects of the same underlying disease process. Although the CRCI does not measure a disease process but instead a CHW intervention, the principles are the same. The CRCI is likely to have a high reliability, because its elements are derived from routinely collected source data. Vaccination records and stopping-smoking clinic referrals are, for example, extracted from GP records and screening attendance is extracted from screening clinic records. Its validity depends on the remit of the CHW - in our case, the focus is on improving uptake of screening, vaccination and smoking cessation clinics; however, there are several other areas that are also worthy of evaluation, for example, impact on self-efficacy, health knowledge, community cohesion and so on. In practice, other outcomes should be used alongside this summary CRCI measure and the cost, feasibility and sensitivity to measure effects of the CRCI will all need to be examined.

CHW interventions that aim to increase uptake of services for which families are eligible are quite likely to increase costs; however, it is likely that this will be off-set in the long term by the gains from early disease detection and avoidance of hospitalisation. Uptake of breast- feeding, stopping someone smoking, and early diagnosis of breast cancer will each yield different long-term gains. At a simplistic level, health economists could calculate the cost per relevant unit of increase in the CRCI (for example, a percentage increase in uptake for services for which families are eligible). However, this would not recognise fully the complex nature of the relationship between uptake of services and the potential health gains from each service. The CRCI approach poses challenges for health economists who have traditionally focused on single outcome measures in cost-effectiveness analysis. How a composite measure might be linked to intervention costs needs to be considered.

Modelling the CRCI against a variety of possible scenarios of population, household and clustering characteristics, we have been able to use it to calculate sample sizes for a future cluster RCT (or other comparison study) for this kind of intervention. This adds value to the evaluation of complex interventions for a number of reasons. Firstly, use of the CRCI as a primary outcome is likely to result in increased statistical power over consideration of any one individual screening/immunisation programme alone. Another merit is that it supports the overall purpose of the intervention, that is to have a wide focus in their activities, because the CRCI does not single out any one service above others in their importance. Narrowly defined primary outcome measures distort complex interventions in RCT contexts. The CRCI may be somewhat harder to interpret, since different elements are incorporated within it; however, to mitigate this, uptake rates for the individual components parts of it can be reported alongside the total CRCI, to show which individual changes make up any CRCI changes. They could be reported with confidence intervals, but without individual testing for significance, since these tests would generally be underpowered.

In our simulation exercise, we found that 85 to 89% of all households will have at least one person who is eligible for a service and, therefore, could be included in the CRCI calculation. Subject to the data being able to be collected from routine sources (possible in all but chlamydia screening which we have therefore excluded from our definition of CRCI) the CRCI can be calculated in a meaningful way and, because it is a comparison of proportions, poses little statistical complexity in terms of its analysis. The CRCI is dependent on the availability and completeness of secondary data - but this is becoming increasingly more accessible though linked datasets such as SAIL or CPRD [43]. In this example, screening records and vaccination records can be matched to individual household members, for use in evaluation with all data, whether in the intervention or control groups, derived from routinely collected sources. If a similar evaluation were proposed in a country where it is not possible to do such matching, this CRCI tool could still potentially be used on a large regional basis, with CHWs introduced into some regions and not others. For this purpose, CRCI would need to be measured at regional level rather than at household level.

Even allowing for clustering at the household level, the CRCI could be a suitable primary outcome score - sample sizes required to demonstrate even small changes in the CRCI between the control and intervention groups are within practical limits, that is around 60 CHWs would be needed to show a 10% increase in the CRCI even if households and clusters are correlated to a plausible degree (with an intra-CHW ICC of 0.02). Brazil has pursued a national policy to scale and integrate CHWs into all primary care teams and now employs over 250,000 CHWs throughout the country. It has seen a corresponding decrease in hospitalisations for primary care conditions, infant mortality, and horizontal inequity [15-20]. In our cluster RCT, we would only need to recruit around a dozen large general practices for an adequately powered study.

Individuals, rather than households, are generally considered to be units of analysis in clinical and health services research. However, considering the likely clustering of disease risk factors and health-seeking behaviours, and also the type of intervention that we are proposing, in complex interventions such as this it may be advantageous to consider the household as a unit of analysis. Our calculations show that around 40% of households of England and Wales will contain members that are eligible for 3 or more services. We also found that the proportion of the simulated population that has taken up a service that it is eligible for is as low as 56 to 60%. This suggests that current strategies to signpost towards available services are inadequate, but equally that intervening at the household level, as opposed to targeting individuals, may be an efficient approach to improving uptake.

Although the CRCI covers over a dozen different nationally available services, there may be other benefits to the CHW service not captured by the CRCI such as mental health improvements or reductions in GP consultations or hospital referrals. Nonetheless, we think the CRCI is a useful first step towards a process-centred composite score of practical value in complex community-based interventions. It may be useful in the UK and in other countries, where its composition would depend on which screening strategies and immunisation services are provided in the relevant country.

## Conclusion

The modelling described indicates that a composite process outcome indicator may have some validity in the context of a complex intervention. The indicator is straightforward in terms of data collection and analysis and could potentially be utilised to measure other complex interventions or be adapted to include specific services depending on the context. Composite outcome scores are useful to increase the power of the statistical analysis around an intervention. For interventions that are not amenable to traditional composites, such as all-cause mortality, the CRCI described here, might be a useful alternative by measuring processes, such as service utilisation, rather than clinical outcomes.

## Abbreviations

CRCI: Composite Referral Completion Indicator
CHW: Community Health Worker
ICC: intra-class correlation coefficient
NHS: National Health Service
RCT: Randomised controlled trial
WHO: World Health Organisation
